# Inhibition of glutamate-carboxypeptidase-II in dorsolateral prefrontal cortex: potential therapeutic target for neuroinflammatory cognitive disorders

**DOI:** 10.1038/s41380-022-01656-x

**Published:** 2022-06-22

**Authors:** Shengtao Yang, Dibyadeep Datta, Elizabeth Woo, Alvaro Duque, Yury M. Morozov, Jon Arellano, Barbara S. Slusher, Min Wang, Amy F. T. Arnsten

**Affiliations:** 1 Department Neuroscience, Yale University School of Medicine, New Haven, CT USA; 2 Department Psychiatry, Yale University School of Medicine, New Haven, CT USA; 3 Department Neurology and Johns Hopkins Drug Discovery, Johns Hopkins School of Medicine, Baltimore, MD USA

**Keywords:** Neuroscience, Drug discovery

## Abstract

Glutamate carboxypeptidase-II (GCPII) expression in brain is increased by inflammation, e.g. by COVID19 infection, where it reduces NAAG stimulation of metabotropic glutamate receptor type 3 (mGluR3). GCPII-mGluR3 signaling is increasingly linked to higher cognition, as genetic alterations that weaken mGluR3 or increase GCPII signaling are associated with impaired cognition in humans. Recent evidence from macaque dorsolateral prefrontal cortex (dlPFC) shows that mGluR3 are expressed on dendritic spines, where they regulate cAMP-PKA opening of potassium (K^+^) channels to enhance neuronal firing during working memory. However, little is known about GCPII expression and function in the primate dlPFC, despite its relevance to inflammatory disorders. The present study used multiple label immunofluorescence and immunoelectron microscopy to localize GCPII in aging macaque dlPFC, and examined the effects of GCPII inhibition on dlPFC neuronal physiology and working memory function. GCPII was observed in astrocytes as expected, but also on neurons, including extensive expression in dendritic spines. Recordings in dlPFC from aged monkeys performing a working memory task found that iontophoresis of the GCPII inhibitors 2-MPPA or 2-PMPA markedly increased working memory-related neuronal firing and spatial tuning, enhancing neural representations. These beneficial effects were reversed by an mGluR2/3 antagonist, or by a cAMP-PKA activator, consistent with mGluR3 inhibition of cAMP-PKA-K^+^ channel signaling. Systemic administration of the brain penetrant inhibitor, 2-MPPA, significantly improved working memory performance without apparent side effects, with largest effects in the oldest monkeys. Taken together, these data endorse GCPII inhibition as a potential strategy for treating cognitive disorders associated with aging and/or neuroinflammation.

## Introduction

**Fig. 1:**
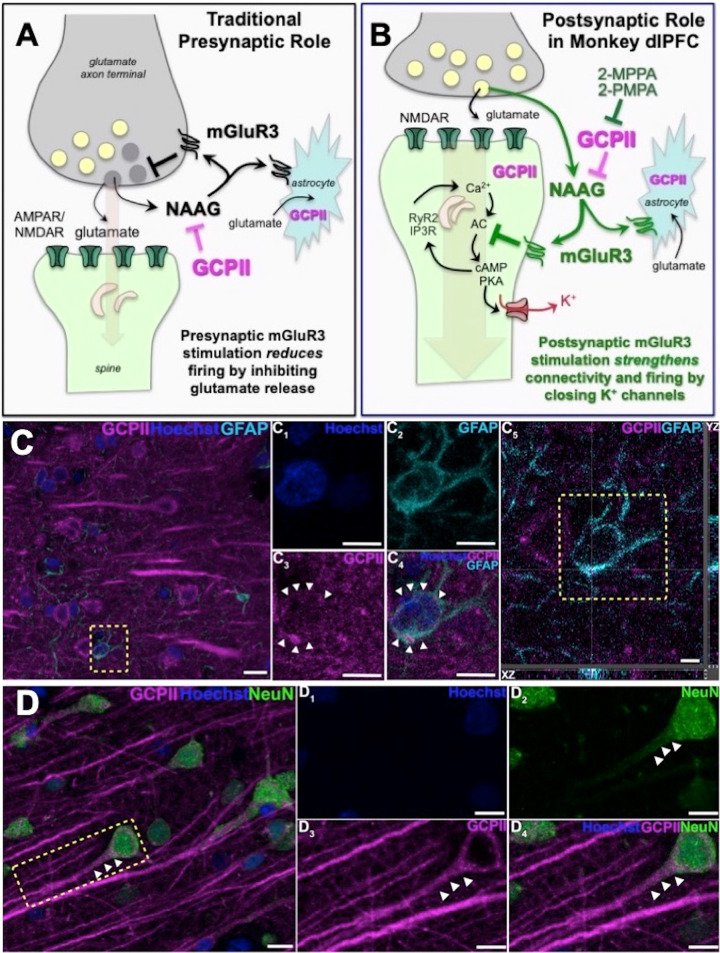
GCPII-NAAG-mGluR3 signaling in primate dlPFC. **A** Traditional view of mGluR3 signaling in a glutamate synapse, where receptors are predominately on presynaptic terminals and on astrocytes, where they reduce glutamate signaling by inhibiting release and increase uptake, respectively. NAAG is co-released with glutamate, and is selective for mGluR3; GCPII catabolism of NAAG would increase neuronal firing in these traditional circuits. **B** GCPII-NAAG-mGluR3 signaling in layer III of the primate dlPFC, where mGluR3 are not presynaptic, but rather are post-synaptic on spines, and NAAG stimulation of mGluR3 inhibits cAMP-K^+^ channel signaling to increase synaptic efficacy and enhance neuronal firing. Thus, GCPII catabolism of NAAG decreases neuronal firing in these recently evolved circuits. **C** Multiple label immunofluorescence showing GCPII labeling (magenta) in its traditional localization in astrocytes which are co-labeled with GFAP (cyan); Hoescht (blue) labels nuclei of all cells. One representative astrocyte is outlined by the yellow dashed box in (C. C_1_-C_4_) correspond to the boxed area in (**C**). In (C_1_–C_4_) GCPII labeling clearly outlines the GFAP positive astrocyte soma, as demarcated by the white arrowheads, (C_5_) Orthogonal sectioning of this region (corresponding region of interest from C outlined by the yellow box). Selected Z-stack image shows co-localization of GCPII and GFAP (magenta and cyan) across three different planes for one point, as indicated by the crossed dashed lines. The right-side bar demonstrates labeling in the YZ plane, while the bottom bar represents labeling in the XZ plane. **D** GCPII is also co-localized in neurons (NeuN, green) with a pyramidal cell-like morphology. White arrowheads highlight GCPII labeling in an apical dendrite. Scale bars: (C,D) 10 μm; (C_1_-C_4_) 5 μm; (C_5_) 2.5 μm; (D_1_-D_4_) 10 μm.

Metabotropic glutamate receptor type 3 (mGluR3s; encoded by *GRM3*) signaling is increasingly linked to higher cognition in humans. mGluR3s differ from other glutamate receptors in that they are stimulated not only by glutamate, but also by NAAG (N-acetylaspartylglutamate), which is co-released with glutamate [1, 2], (as well as with other neurotransmitters [3], e.g. acetylcholine [4]), and is selective for mGluR3 [5–7] (Fig. 1A, B). NAAG is catabolized by glutamate carboxypeptidase II (GCPII), a zinc metalloenzyme that is increased under neuroinflammatory conditions [8–10], reducing mGluR3 signaling. GCPII is also robustly increased in the intestinal mucosa of patients with inflammatory bowel disease [11, 12], and a recent paper has shown a similar large elevation in GCPII expression in the brains of patients who died with severe COVID19 [13]. GCPII is known by many other names [14], including folate hydrolase (thus encoding by the *FOLH1* gene), and prostate-specific membrane antigen (PSMA), due to its high levels of expression in the prostate, with increased expression in prostate cancer. GCPII/PSMA levels in plasma can serve as an early diagnostic of prostate cancer, as GCPII in the prostate is endocytosed from the extracellular space into endosomes, which fuse with multivesicular bodies to form and secrete exosomes [15, 16]. However, GCPII-mGluR3 also appears to play a key role in human cognition.

Both biochemical and genetic studies have linked reductions in mGluR3 signaling to cognitive deficits and increased risk of mental disorders [17]. For example, alterations in *GRM3* are a GWAS-validated risk factor for schizophrenia [18] and mGluR3 levels are decreased in the dorsolateral prefrontal cortex (dlPFC) of patients with schizophrenia [19], the cortical region needed for working memory, attention regulation and higher cognition [20–22]. NAAG levels are also decreased in vulnerable brain regions in patients with Alzheimer’s Disease (AD) [23–25], and reduced NAAG levels correlated with cognitive deficits in multiple sclerosis [26]. Conversely, GCPII levels are increased in the dlPFC of patients with schizophrenia [19], and gain-of-function mutations in *FOLH1* that reduce NAAG-mGluR3 signaling are linked to inefficient cognition and lower scores on IQ tests in healthy subjects [27]. These findings emphasize that mGluR3-GCPII signaling is more important to higher cortical functioning in humans than previously assumed, and thus is an important focus for research.

Traditionally, both mGluR3 and GCPII have been localized on astrocytes, where mGluR3 enhance glutamate uptake (Fig. 1A, B; [28]). It has been widely thought that mRNA for GCPII is selectively localized in astrocytes and not in other cell types in brain [29], although recent work has identified GCPII message in neurons [30]. In contrast, mGluR3s have been localized on both astrocytes and neurons, typically on presynaptic terminals [31–33], where they inhibit glutamate release (Fig. 1A) e.g. in spinal cord pain circuits [34–36]. Importantly, mGluR3 are predominately localized on presynaptic terminals in the rodent medial PFC, with only a small proportion on spines in layer II/III [37]. In contrast, recent research has shown that mGluR3 are predominately post-synaptic rather than presynaptic in layer III of the primate dlPFC, and play a key role in enhancing working memory [38]. This work has shown that mGluR3 are localized on dendritic spines in layer III of macaque dlPFC, the layer that contains the recurrent excitatory microcircuits that generate the persistent neuronal firing needed to maintain information “in mind”. As illustrated in Fig. 1B, mGluR3 strengthen synaptic connectivity and increase working memory-related neuronal firing by inhibiting cAMP opening of K^+^ channels on spines [17, 38]. Specifically, cAMP reduces persistent firing by increasing the open state of HCN1/2 channels [39] which in turn open Slack K^+^ channels [40], while PKA reduces persistent firing by increasing the open state of KCNQ2 channels [41]. Increased opening of these K^+^ channels contributes to loss of dlPFC persistent firing with advancing age, due to reduced regulation of cAMP-PKA signaling [42, 43]. Thus, restoration of regulation of cAMP-PKA signaling with increased mGluR3 actions may help to restore the layer III dlPFC neuronal firing needed for sustained mental representations. As layer III dlPFC is a focus of pathology in both schizophrenia [44] and AD [45], and dlPFC is also a focus of cognitive deficits in multiple sclerosis [46], this mechanism has particular clinical relevance, suggesting that elevated GCPII in dlPFC may contribute to dlPFC dysfunction in inflammatory disorders by eroding beneficial mGluR3 signaling. Although GCPII inhibition has therapeutic potential for the treatment of cognitive disorders, the subcellular expression of GCPII in layer III of the primate dlPFC and the effects of GCPII inhibition on higher cognition in nonhuman primates are not known.

The current study examined GCPII expression and function in primate dlPFC, localizing GCPII at the cellular and ultrastructural levels. Given the therapeutic potential of GCPII inhibition for cognitive disorders, the current study also examined the effects of local vs. systemic GCPII inhibition on dlPFC neuronal firing and working memory performance, respectively, in aged rhesus monkeys. The study utilized two GCPII inhibitors: (1) 2-MPPA (also known as GPI-5693, IC_50_ = 90 nM) which is orally active, has modest brain penetrance, and has been tested in humans [47, 48], and (2) 2-PMPA, which has higher affinity for GCPII (IC_50_ = 0.3 nM) but very limited brain penetrance [49]. The results show that GCPII has extensive neuronal as well as astrocytic expression in aged monkey dlPFC, and that GCPII inhibitors markedly enhance dlPFC neuronal firing, with important potential as cognitive therapeutics when they can gain entry into brain.

## Materials and methods

All research was approved by the Yale IACUC and was in accordance with NIH regulations.

### Subjects

The physiological or behavioral studies utilized rhesus monkeys from the Yale colony; monkeys were pair-housed and received daily environmental enrichment, including fresh fruits and vegetables. Animals were water- or food-regulated for physiological and behavioral studies, respectively, receiving their full rations after their testing session. The immunofluorescence and immunoelectron microscopy studies used brain tissue from our brain bank. Details regarding sex and ages of the monkeys are provided in each section below.

### Antibodies

We used the mouse PSMA/FOLH1/NAALADase I/GCPII antibody raised against Lys44-Ala750 which contains the extracellular domain of GCPII (MAB4234; R&D Systems; 1:50). The antibody recognizes a very specific band for GCPII by western blotting with no other non-specific bands. The recognition of PSMA/FOLH1/GCPII was confirmed using antigen-down ELISA (R&D Systems). In direct ELISA’s, the antibody shows less than 10% cross-reactivity with rhNAALADase-like 2 and no cross-reactivity with rhNAALADase-like 1, rhNAALADase-like 3, rmNAALADase I, or rmNAALAase-like 2. The multiple label immunofluorescence used the following additional antibodies: GFAP (1:500, BioLegend, Cat# 829401), NeuN (1:300, EMD Millipore, Cat# ABN78), and Iba1/AIF-1 (1:300, Cell Signaling Tech, Cat# 17198 T).

### Multiple label immunofluorescence with super resolution confocal microscopy

This work utilized fixed tissue from our existing brain bank of 2 female rhesus monkeys aged 24 and 31 years. Immunofluorescence staining was carried out on free-floating sections. Antigen retrieval was performed with 2x Antigen Unmasking Solution Citrate Buffer pH 6.0 (Vector Laboratories, H-3300-250) in a steam cooker for 40 minutes at high temperature. The free-floating sections were left to cool for 15 min at RT, as stated in the manufacturer’s instructions. After washing the sections in deionized water (1 × 5 min), followed by tap water (2 × 5 min), they were transferred to 1X TBS for 10 min. Sections were blocked for 1 h at RT in 1X TBS containing 5% bovine serum albumin, 2% Triton X-100, and 10% normal goat serum. Sections were incubated for 48 h at 4 °C with specific primary antibodies (see dilutions above), followed by incubation overnight at 4 °C with secondary antibodies (1:1000, Alexa-Fluor conjugated, Invitrogen). Following incubation in secondary antibodies, they were incubated in 70% Ethanol with 0.3% Sudan Black B (MP Biomedicals, Cat# 4197-25-5), to decrease autofluorescence from lipofuscin. The sections were then washed in 0.02% Tween in 1X TBS (3 × 5 minutes) followed by wash in 1X TBS (1 × 5 min) and counterstained with Hoechst 33342 for 10 min (1:10,000, ThermoFisher, Cat# H3570). The sections were washed in 1X TBS (pH 7.4, 3 × 10 min) before mounting onto slides using ProLong Gold Antifade Mountant (Invitrogen, Cat# P36930).

Confocal images were acquired using a Leica TCS SP8 Gated STED 3X super resolution microscope, with the HC PL APO 100X/1.40 oil white objective (Leica) and HCX PL APO CS 63X/1.40 oil white objective (Leica). Z-stacks were obtained with 0.3-μm steps under laser excitation at 407 nm, 488 nm, 543 nm, and 633 nm. Emission filter bandwidths and sequential scanning acquisition were set up in order to avoid possible spectral overlap between fluorophores. The confocal Z-stacks were processed into maximum intensity Z-projections using Fiji and background subtraction with a rolling ball radius of 50 pixels and 100 pixels was applied to all 63X and 100X channels, respectively (applied to the entire panel). Images were labeled and assembled into a figure using Adobe Photoshop CS5 Extended (version 12.0.4 ×64, Adobe Systems Incorporated). To confirm double immunofluorescence co-localization, the acquired z-stacks for all antibody combinations were examined using the orthogonal sectioning function on the Leica LASX software. For one point of interest, three planes of view can be examined (XY, YZ, and XZ planes). See [50] for additional methodological details.

### ImmunoEM

This work utilized fixed tissue from our brain bank of 2 female rhesus monkeys aged 28 and 30 years, who had been perfused transcardially with 100 mM phosphate buffer (PB), followed by 4% paraformaldehyde, 0.05% glutaraldehyde in 100 mM PB. Following perfusion, a craniotomy was performed, and the entire brain was removed and dissected, including a frontal block containing the primary region of interest surrounding the principal sulcus. The brains were sectioned coronally at 60 μm on a vibratome (Leica) across the entire rostrocaudal extent of the dlPFC. The sections were cryoprotected through increasing concentrations of sucrose solution (10%, 20% and 30% each overnight), cooled rapidly using liquid nitrogen and stored at –80 °C. To enable penetration of immunoreagents, all sections went through 3 freeze-thaw cycles in liquid nitrogen. As described previously for immunocytochemistry [38], sections were incubated for 72 hr at 4 °C with primary antibody for GCPII in Tris-buffered saline (TBS), and transferred for 2 hr at room temperature to species-specific biotinylated Fab’ or F(ab’)_2_ fragments in 50 mM TBS. Non-specific reactivity was suppressed with 10% normal goat serum (NGS) and 2% bovine serum albumin (BSA), and antibody penetration was enhanced with 0.3% Triton X-100 in 50 mM TBS. To reveal immunoperoxidase labeling, sections were incubated with the avidin-biotin peroxidase complex (ABC) (1:300; Vector Laboratories) and then visualized in 0.025% Ni-intensified 3,3-diaminobenzidine tetrahydrochloride (DAB; Sigma Aldrich,) as a chromogen in 100 mM PB with the addition of 0.005% hydrogen peroxide for 12 min. Sections were then exposed to osmification, dehydration and standard resin embedding following typical immunoEM procedures. Omission of primary antibodies or substitution with non-immune serum resulted in complete lack of immunoperoxidase labeling. For electron microscopy, blocks containing dlPFC layer III were sampled and mounted onto resin blocks. The specimens were cut into 50 nm sections using an ultramicrotome (Leica) and analyzed under a FEI Tecnai Biotwin transmission electron microscope at 80 kV. Structures were digitally captured at ~x25,000-x100,000 magnification with a SIS Morada 11 megapixel CCD camera.

### Pharmacological agents

2-MPPA was synthesized according to published procedures [47] at Johns Hopkins Drug Discovery. 2-PMPA was purchased from Redicius s.r.o. (Uničov, Czech Republic).

### Iontophoresis coupled with single unit recordings

Physiology was conducted in two rhesus monkeys, one middle-aged male (14 years) and one aged female (24 years) performing the oculomotor delayed response (ODR) test of spatial working memory described in the Results below. Single-unit recordings of Delay cells were conducted at the caudal principal sulcus in dlPFC. The GCPII inhibitors 2-MPPA or 2-PMPA were applied via iontophoresis; small electrical currents (5-100 nA) delivered minute amounts of drug to the recording site insufficient to alter behavior. d’ was calculated as a measure of the strength of spatial tuning using the formula:

$$d^{\prime} = (mean_{pref} - mean_{nonpref})/\sqrt {(sd_{pref}^2 + sd_{nonpref}^2)/2} .$$

### GCPII inhibitor effects on working memory performance

The effects of systemic administration of 2-MPPA or 2-PMPA on spatial working memory were tested in *n* = 7 (2 male, 5 female) aged monkeys ranging in age from 21 to 32 years. The monkeys had been pretrained on the variable delay, spatial delayed response task in a Wisconsin General Test Apparatus (WGTA) as illustrated in Supplementary Fig. 1. As chair and head restraint are often contraindicated in very old monkeys for health reasons, and as aged monkeys can be reticent to interact with a computer monitor, the WGTA version of the task is more appropriate for most aged animals than the ODR task used for physiology. In the WGTA version of the task, the monkey watches as the experimenter bait one of two wells with a food reward, the wells are then covered with identical plaques and a screen is lowered for a prescribed delay. After the delay period is over, the screen is raised and the monkey must choose based on its memory of the location of the baited well. The spatial position of the reward randomly changes over the 30 trials that make up a daily test session, and the monkey must constantly update the contents of working memory to perform correctly. Performance of this task depends on the integrity of the dlPFC [51, 52], and the persistent firing of neurons within the dlPFC [53]. In this study, variable delays were used, ranging from 0 s to the delay that produced chance performance, and were adjusted to produce overall performance of ~70% correct, thus leaving room for either improvement or impairment in performance. The monkeys were tested twice a week, by an experimenter who was blind to drug treatment conditions but highly familiar with the normative behavior of each monkey. In addition to performance on the task, they were rated for potential changes in sedation, agitation or aggression.

2-MPPA or 2-PMPA were diluted in sterile saline immediately before use and administered i.m. or p.o. 2 h before testing. Performance on drug was compared to the monkeys’ own performance on vehicle. It is noteworthy that 2-MPPA breaks down when in solution, even if frozen, and thus all solutions were made fresh and used immediately. Monkeys were required to return to stable baseline performance for at least 2 successive test sessions prior to receiving another dose of drug or vehicle; thus there was at least a 10 day washout period between doses. Prior to this study the monkeys had been tested on acute, low dose treatment with a muscarinic M1 receptor positive allosteric modulator. However, as noted, long washout periods are always employed between doses, and monkeys are required to return to stable baseline performance prior to additional drug testing in order to ensure that the effects of any previous drug treatment have been minimized. Given the long washout periods, this study of GCPII inhibitors took more than 2 years.

### Statistics

Drug effects on neuronal firing were tested with repeated one-way or two-way ANOVA, and 2-tailed paired-samples *t* test. Behavioral data were analyzed using repeated measures ANOVA with paired contrasts. For systemic drug administration, we analyzed the 0, 0.01, 0.1, and 1.0 mg/kg doses and compared each dose to vehicle. Pearson’s r test was used for correlations with age. All statistics were two-sided. Statistics were performed with SPSS software. For detailed methods, see [38].

## Results

### Immunofluorescence and ImmunoEM of rhesus monkey layer III dlPFC

**Fig. 2:**
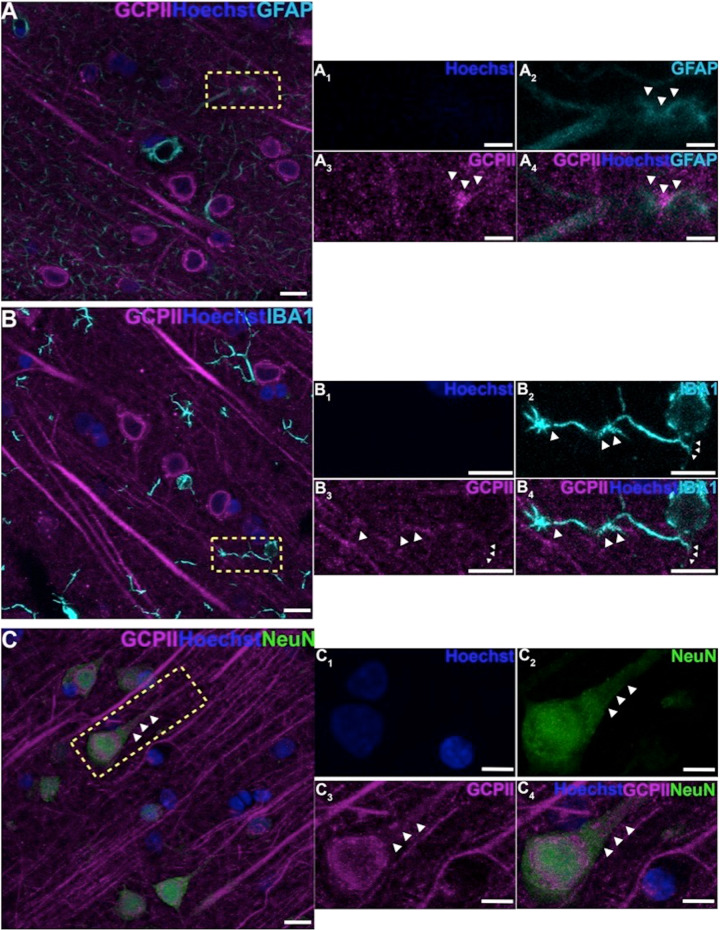
GCPII cellular labeling in layer III of the aged monkey dlPFC using multiple label immunofluorescence. **A** Another example of GCPII labeling (magenta) in astrocytes that are co-labeled with GFAP (cyan). (A_1_-A_4_) correspond to the yellow boxed region in (**A**). White arrowheads demarcate co-localization of GFAP and GCPII. **B** GCPII is occasionally observed in the processes of microglia, labeled with iba1 (cyan), and outlined by the white arrowheads. **C** Another example of GCPII co-localized in neurons (NeuN, green) with a pyramidal cell-like morphology. White arrowheads highlight GCPII labeling in an apical dendrite. Hoechst label (blue) indicates nuclei. (B_1_-B_4_), and (C_1_-C_4_) magnified regions correspond to the areas delineated by the yellow boxes in (**B**) and (**C**). Scale bars: (**A**–**C**) 10 μm; (A_1_-A_4_) 2.5 μm; (B_1_-B_4_) 5μm; (C_1_-C_4_) 5 μm.

Multiple label immunofluorescence (MLIF) demonstrated GCPII glial expression in astrocytes as expected (Figs. 1C, 2A), consistent with the immunoEM described below. There was also rare GCPII expression in microglial processes (Fig. 2B); the absence of labeling in the cell bodies of microglia, and the relatively crude resolution of light microscopy made it difficult to observe the true degree of co-localization in microglial processes. However, the MLIF also showed extensive GCPII expression in neurons in aged monkey dlPFC (Figs. 1D, 2C). Neurons co-labeled with NeuN and GCPII had a pyramidal cell-like morphology, with GCPII prominent in apical dendrites (Figs. 1D, 2C). DAB immunoperoxidase labeling (Supplementary Fig. 2A, B), and immunoEM (Supplementary Fig. 2C) confirmed the presence of GCPII in the pyramidal cell soma and apical dendrite; GCPII could also be seen in pyramidal-like cells in other layers (Supplementary Fig. 2D).

**Fig. 3:**
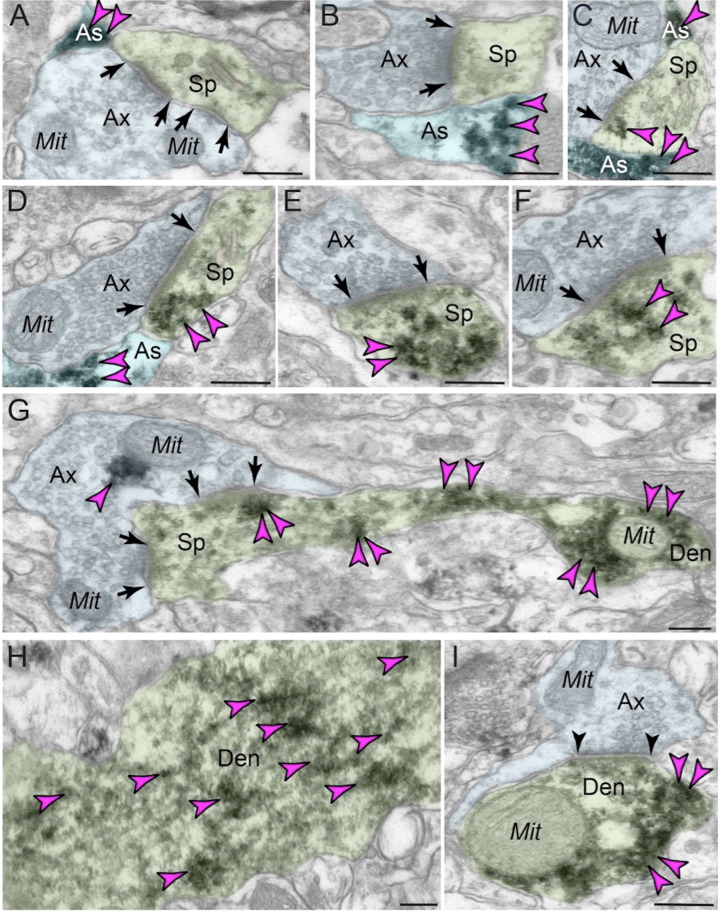
ImmunoEM subcellular localization of GCPII in aged macaque dlPFC layer III. **A**–**C** Immunoperoxidase labeling for GCPII in aged macaque (28–30 y) dlPFC layer III is visualized within astroglial leaflets ensheathing asymmetric (likely glutamatergic) synapses. GCPII immunolabeling appears to show a preference for the astroglial leaflets (PAPs) near the plasma membrane in perisynaptic locations near the excitatory glutamatergic synapse. **D**–**G** GCPII immunolabeling is prominently expressed in *postsynaptic* compartments in dendritic spines, near the plasma membrane and PSD synaptic active zone (**D, F** and **G**). Intriguingly, GCPII immunolabeling is visualized in vesicular-like structures within the dendritic spine head, positioned to undergo endocytosis or exocytosis (**E** and **F**). In (**G**), a dendritic spine is seen emanating from the dendritic shaft of a pyramidal cell; both are immunopositive for GCPII. Sparse presynaptic GCPII labeling is also observed in the axon terminal in association with mitochondria. All dendritic spines receive axospinous Type I asymmetric glutamatergic-like synapses, and the spine apparatus is pseudocolored in pink in the spine head. Synapses are between arrows. **H–I** In aged macaque dlPFC layer III, GCPII immunolabeling was visualized in dendritic shafts and was associated with microtubules oriented in parallel bundles. In (**I**), the dendritic shaft receives an axodendritic symmetric Type II synapse (arrowheads) and GCPII immunolabeling within the dendritic shaft was in close proximity to mitochondria. Color-coded arrowheads (magenta) point to GCPII immunoreactivity. Profiles are pseudocolored for clarity. Ax axon, Mit mitochondria, Sp dendritic spine, As astroglia, Den dendrite. Scale bars: 200 nm.

ImmunoEM of layer III dlPFC verified GCPII localization in glia, frequently in perisynaptic astrocytic processes (PAPs) next to glutamate-like asymmetric synapses, positioned to regulate glutamate signaling (Fig. 3A–C; PAPs are too small to see with MLIF). ImmunoEM also documented extensive GCPII neuronal labeling in dendritic spines (Fig. 3D–G), often near the plasma membrane (Fig. 3D, E, G). Interestingly, GCPII appeared in round structures in dendritic spines (e.g. Fig. 3E, F), as well as in some astrocytes (Fig. 3B). These data could be consistent with exosomal release from astrocytes to the extracellular space, and endosomal uptake by pyramidal cell spines and dendrites. As spines and astrocytes are the major sites for mGluR3 localization in layer III dlPFC [38], GCPII localization at these same sites may directly influence the degree of NAAG-mGluR3 signaling. ImmunoEM also showed GCPII labeling of dendrites (Fig. 3G–I), often on microtubules (Fig. 3H), suggesting that GCPII may traffic within the neuron.

In summary, GCPII was expressed in astroglial processes as expected, but was also evident in pyramidal cells, where the pattern of labeling in dendritic spines would be consistent with GCPII uptake via endocytosis.

### Physiology

**Fig. 4:**
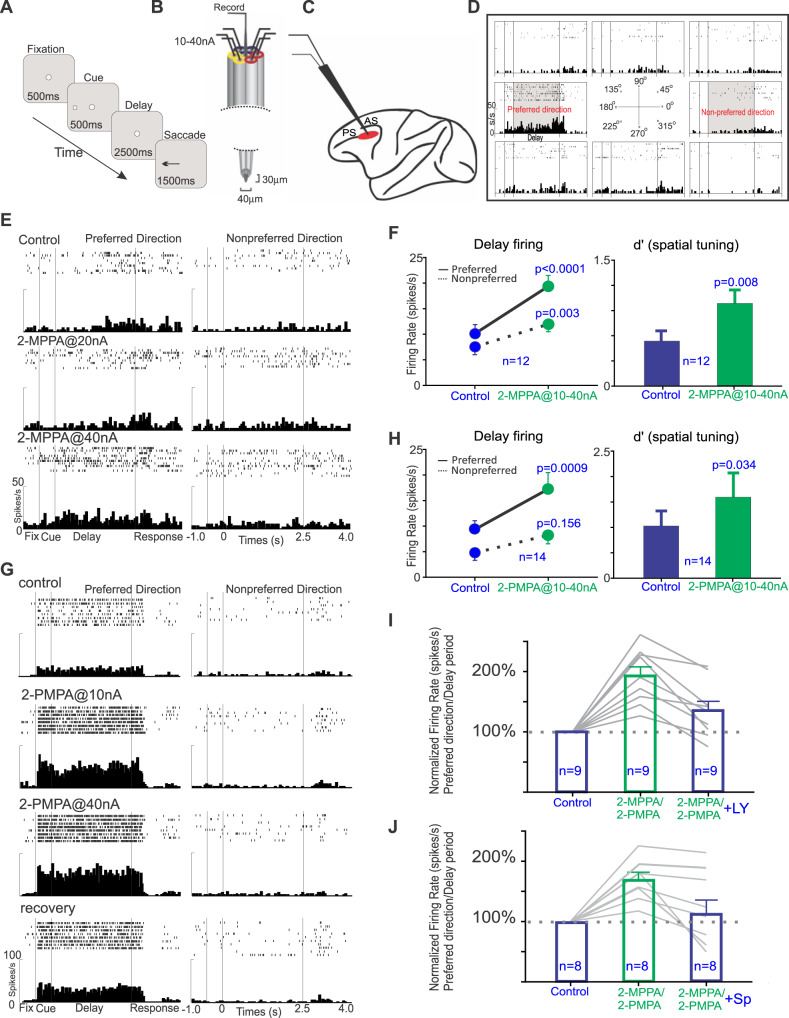
The effects of iontophoretic application of GCPII inhibitors onto dlPFC Delay cells in aged monkeys performing an oculomotor delayed response task. **A** The oculomotor delayed response task. **B** The iontophoretic recording electrode. **C** The recording site in dlPFC; PS = principal sulcus; AS = arcuate sulcus. **D** An example of a dlPFC Delay cell, with spatially-tuned persistent firing for its preferred direction (180°). **E** An example of the effects of iontophoresis of 2-MPPA onto a dlPFC Delay cell in an aged female monkey with weak task-related firing under control conditions. **F** the average effects of 2-MPPA on dlPFC delay-related firing in Delay cells from both monkeys. **G** An example of a dlPFC Delay cell whose firing for its preferred direction is greatly increased by 2-PMPA in a middle-aged male monkey. **H** population response for 2-PMPA. **I** The enhancing effects of 2-MPPA or 2-PMPA were significantly reversed by co-application of the mGluR2/3 antagonist, LY341495. **J** The enhancing effects of 2-MPPA or 2-PMPA were significantly reversed by co-application of the PKA activator, Sp-cAMPS. Note that Sp-cAMPS activates PKA signaling, but is downstream from cAMP-HCN channel signaling, and thus may have only partial actions in reversing the beneficial effects of NAAG-mGluR3 signaling.

The effects of iontophoretic application of the GCPII inhibitors, 2-MPPA or 2-PMPA, directly onto dlPFC Delay cells were assessed in a middle-aged and aged monkey performing an oculomotor visuospatial working memory (ODR) task (Fig. 4A). In this task, the monkey fixates on a central point for 0.5 s to initiate a trial; a cue then comes on for 0.5 s in one of eight locations. The monkey must remember the spatial location over a delay period of 2.5 s. At the end of the delay, the fixation point is extinguished, and the monkey can move its eyes to the remembered location within 1 s for juice reward. Single unit recordings coupled with iontophoresis (Fig. 4B) were made from the dlPFC (Fig. 4C), with focus on the Delay cells with spatially-tuned, delay-related firing that underlie working memory (Fig. 4D). Delay-related firing naturally decreases with increasing age, starting in middle age, due to excessive cAMP-PKA-K^+^ channel signaling [42]. The current study tested whether inhibiting GCPII to increase mGluR3 regulation of cAMP-PKA-K^+^ channel signaling would enhance delay-related firing in the aging monkeys.

As shown in Fig. 4, both compounds significantly enhanced delay-related neuronal firing. An example of a Delay cell treated with 2-MPPA is shown in Fig. 4E; this neuron from the aged monkey had low firing under control conditions, and showed a significant, dose-related increase in delay-related firing for the neuron’s preferred direction (Fig. 4E; control vs. 2-MPPA@40 nA condition: two-way ANOVA, F_directionxdrug_(1,30) = 4.868, *p* = 0.0352; F_drug_(1,30) = 7.991, *p* = 0.0083; F_direction_ (1,30) = 13.71, *p* = 0.0009; Sidak’s multiple comparisons: preferred direction, *p* = 0.0025 and non-preferred direction, *p* = 0.8871). The average response of all Delay cells (*n* = 12) to 2-MPPA is shown in Fig. 4F; 2-MPPA significantly increased delay-related firing for the neurons’ preferred direction, with lesser enhancement for nonpreferred directions, thus significantly enhancing the d’ measure of spatial tuning (Fig. 4F; delay firing: R-two-way ANOVA, F_directionxdrug_(1, 11) = 13.95, *p* = 0.0033; Sidak’s multiple comparisons: preferred direction, *p* < 0.0001 and non-preferred direction, *p* = 0.0027; d’: control vs 2-MPPA, t(11) = 3.239, *p* = 0.0079, two-tailed paired *t* test). An example of a Delay cell from the middle-aged monkey treated with 2-PMPA is shown in Fig. 4G; this neuron had a large increase in delay-related firing even at the lowest dose (Fig. 4G; control vs. 2-PMPA@10 nA condition: two-way ANOVA, F_directionxdrug_(1,39) = 26.22, *p* < 0.0001; F_drug_(1,39) = 27.48, *p* < 0.0001; F_direction_ (1,39) = 271.8, *p* < 0.0001; Sidak’s multiple comparisons: preferred direction, *p* < 0.0001 and non-preferred direction, *p* = 0.9999); firing returned towards baseline when drug was no longer applied (recovery). The average of 14 neurons to 2-PMPA is shown in Fig. 4H; 2-PMPA significantly enhanced delay-related firing for the neurons’ preferred direction and significantly improved d’ spatial tuning (Fig. 4H; delay firing: R-two-way ANOVA, F_directionxdrug_(1, 13) = 6.576, *p* = 0.0235; Sidak’s multiple comparisons: preferred direction, *p* = 0.0009 and non-preferred direction, *p* = 0.156; d’: control vs 2-PMPA, t(13) = 2.366, *p* = 0.0342, two-tailed paired *t* test).

As most Delay cells also fire to the cue and in anticipation of the eye movement response, further analyses examined drug effects on the firing of Delay cells during the cue, delay and response epochs of the task, as well as during initial fixation to initiate a trial, and during the intertrial interval (ITI). As shown in Supplementary Fig. 3A, 2-MPPA significantly increased firing for the cue, delay and response epochs, but did not significantly alter firing during the ITI or fixation. There was a trend for enhancement during the initial fixation. A similar pattern could be seen with 2-PMPA (Supplementary Fig. 3B). Thus, both compounds increased the task-related firing of Delay cells, without altering firing during the intertribal intervals.

We next tested the hypothesis that GCPII inhibition increased delay-related firing by enhancing mGluR3 regulation of cAMP-PKA-K^+^ signaling (Fig. 1B). As there are no electrically charged mGluR3 antagonists, we tested for actions at mGluR3 using the mixed mGluR2/3 antagonist, LY341495. LY341495 significantly reversed the enhancement in delay-related firing (Fig. 4I: preferred direction: Repeated one-way ANOVA, F(1.812, 14.49) = 33.17, *p* < 0.0001; Tukey’s multiple comparisons: control vs. 2-MPPA/PMPA, *p* < 0.0001; control vs. 2-MPPA/PMPA + LY, *p* = 0.0.1058; 2-MPPA/PMPA vs. 2-MPPA/PMPA + LY, *p* = 0.0029), consistent with GCPII inhibition leading to increased NAAG stimulation of mGluR3. We also tested whether the effects of the GCPII inhibitors could be reversed by Sp-cAMPS, which activates PKA-K^+^ signaling. Overall, co-application of Sp-cAMPS with GCPII inhibitors significantly reversed the enhancement in delay-related firing (Fig. 4J; preferred direction: Repeated one-way ANOVA, F(1.376, 11) = 9.266, *p* = 0.0074; Tukey’s multiple comparisons: control vs. 2-MPPA/PMPA, *p* = 0.0004; control vs. 2-MPPA/PMPA + Sp, *p* = 0.7637; 2-MPPA/PMPA vs. 2-MPPA/PMPA + Sp, *p* = 0.0352), consistent with this signaling mechanism. The incomplete reversal in a small subset of neurons may be due to the absence of direct actions on cAMP, which is not activated by Sp-cAMPS.

### Behavior

We examined the effects of acute systemic administration of the GCPII inhibitors 2-MPPA or 2-PMPA on the working memory performance of aged monkeys with naturally-occurring working memory deficits. Results were compared to the monkeys’ own performance following vehicle administration.

**Fig. 5:**
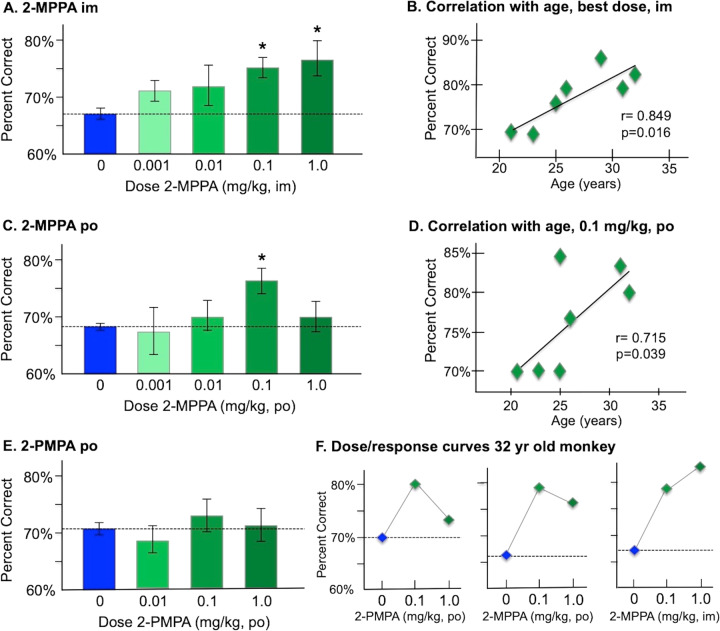
The effects of systemic administration of the GCPII inhibitors 2-MPPA and 2-PMPA on the working memory performance of aged rhesus monkeys. **A** The effects of intramuscular injection (im) of 2-MPPA on working memory performance; results represent mean percent correct ± SEM. **B** The correlation between percent correct on a best dose of 2-MPPA (0.1 or 1.0 mg/kg) during a single test session and age in years for each monkey in the experiment, showing a significant correlation, with older animals showing more benefit than younger animals. **C** The effects of oral administration (po) of 2-MPPA on working memory performance; results represent mean percent correct ± SEM. **D** The correlation between percent correct on 2-MPPA (0.1 mg/kg) during a single test session and age in years for each monkey in the experiment, showing a significant correlation, with older animals showing more benefit than younger animals. **E** The effects of oral administration (po) of 2-PMPA on working memory performance; results represent mean percent correct±SEM. **F** Individual dose/response curves for the oldest (32 years) monkey, showing some benefit with oral 2-PMPA or 2-MPPA, but the greatest benefit with im injection of 2-MPPA. *indicates significantly different from vehicle control.

Intramuscular administration of 2-MPPA two hours prior to testing significantly improved performance compared to vehicle control (F(3,18) = 3.3, *p* = 0.044), with a linear dose/response (F(1,6) = 22.39, *p* = 0.003). Significant improvement was seen at both the 0.1 mg/kg (*p* = 0.005) and 1.0 mg/kg (*p* = 0.016) doses (Fig. 5A). 2-MPPA was more effective in older monkeys, with increased efficacy correlating with increased age (*r* = 0.85, *p* = 0.016; Fig. 5B).

Oral administration of 2-MPPA was less effective (Fig. 5C), with a trend in overall improvement (F(3,18) = 2.48, *p* = 0.094), and enhancement limited to the 0.1 mg/kg dose (*p* = 0.011). The efficacy of 0.1 mg/kg 2-MPPA also significantly correlated with increased age (*r* = 0.715, *p* = 0.039; Fig. 5D), with improvement in the oldest animals (>25 years).

In contrast to 2-MPPA, administration of 2-PMPA (p.o.) had no significant effect on performance (F(3,18) = 0.55, *p* > 0.6; Fig. 5E), likely due to its negligible oral bioavailability and poor brain penetration. There was spotty improvement in some animals, including the oldest monkey (32 yrs; Fig. 5F). However, 2-MPPA had more robust effects than 2-PMPA in this animal, particularly following intramuscular treatment (Fig. 5F).

## Discussion

The current study found extensive localization of GCPII in pyramidal cells as well as in astrocytes (especially PAPs) in layer III of the aged macaque dlPFC. Inhibition of GCPII with iontophoretic application of either 2-MPPA or 2-PMPA directly onto dlPFC neurons markedly enhanced delay-related firing in aging monkeys, demonstrating the importance of this mechanism to dlPFC higher cognition, and the great potential for enhancement. However, improvement with systemic administration was only seen with the more brain penetrant compound, 2-MPPA. Improvement with 2-MPPA correlated with increased age, likely due to the blood brain barrier becoming leakier with older age [54]. Older animals may also benefit more from GCPII inhibition if they have greater inflammation and reduced beneficial NAAG-mGluR3 actions on dendritic spines. Altogether, the data emphasize that GCPII inhibition has great potential as a therapeutic strategy for restoring higher cognitive function.

### Neuronal as well as glial expression of GCPII

It has been widely accepted that GCPII is synthesized and expressed in astrocytes, with mRNA found exclusively in astrocytes and not in other cell types [29]. Similar findings were seen in the white matter from human frontal cortex [55], although long post-mortem intervals may have degraded membranes and expression in gray matter. The current immunofluorescent and immunoEM data from perfusion-fixed macaque dlPFC confirms GCPII astrocytic expression in the monkey, but also provides the first evidence of extensive GCPII protein expression in pyramidal cells in layer III dlPFC. These data are consistent with our recent findings from aged rat medial PFC, where GCPII was also expressed in neurons with a pyramid cell-like morphology [50], and with studies showing GCPII expression in mouse hippocampal pyramidal cells [56]. Neuronal expression of GCPII is also increased following hypoxia in rats, both in vitro and in vivo, [30] suggesting that neuronal expression may vary depending on experimental conditions.

Previous studies prostate cancer cells have shown that GCPII can be endocytosed from the cell surface into endosomes that can fuse with multivesicular bodies to form and secrete exosomes into the extracellular environment [15, 16]. Thus, it is possible that GCPII is synthesized in astrocytes but taken up into nearby neuronal spines and dendrites, especially under inflammatory conditions [30]. Interestingly, NAAG has also been localized within dendritic spines where it is seen in vesicles, and is released into the extracellular space for retrograde mGluR3 signaling [57]. It is possible that GCPII is also released in this manner, e.g. under conditions of inflammation. NAAG levels may provide a metabolic signal to the neuron regarding energy availability. NAAG synthesis requires NAA, and NAA levels are an indicator of mitochondrial function. Thus, NAAG levels may be regulated by mitochondrial pyruvate-NAA metabolism [58, 59], and may signal to the neuron that energy is available to support the persistent firing needed for working memory. In contrast, GCPII release may predominate under inflammatory conditions to decrease NAAG levels, weaken network connectivity and reduce neuronal firing to conserve energy stores.

### Results with 2-MPPA vs. 2PMPA on neuronal firing and cognitive performance

The physiological data revealed that endogenous GCPII expression reduced the dlPFC persistent firing needed for working memory, as neuronal firing was greatly enhanced by local GCPII inhibition. The enhancing effects of GCPII inhibitors were reversed by blocking mGluR3 or PKA signaling, consistent with the newly evolved role of mGluR3 in primate dlPFC, inhibiting cAMP-PKA-K^+^ channel signaling to strengthen network connectivity, enhance persistent firing and improve working memory. These data are consistent with the growing human literature showing that genetic mutations that increase GCPII expression or reduce mGluR3 expression are associated with inefficient dlPFC activity and impaired cognition [27, 60]. The robust enhancement in dlPFC delay-related firing and d’ measures of spatial representation following local GCPII inhibition also indicate that GCPII inhibitors have great therapeutic potential for treating PFC cognitive disorders, and may be particularly useful in those with an inflammatory etiology, as described below. However, the challenge for systemic administration will be facilitating compound access to brain, given that current GCPII inhibitors are all highly charged. The positive cognitive results with systemic administration of 2-MPPA, which has some brain penetrant properties, provide a proof-of-concept that this mechanism is directly relevant to treating higher cognition in primates.

### Relevance to potential treatments for cognitive disorders

mGluR3-NAAG-GCPII signaling is increasingly relevant to human cognition, and to the etiology and treatment of mental disorders, especially cognitive disorders associated with neuroinflammation. The recent finding that COVID19 increases GCPII expression in brain [13] are consistent with the literature showing that inflammation increases GCPII expression in animals [8–10], and that COVID19 can induce prolonged deficits in the executive and working memory functions that rely on the dlPFC [61–64].

Genetic studies also show that mutations that increase GCPII and/or decrease mGluR3 signaling are associated with cognitive deficits. A recent study of both healthy human subjects and patients with psychosis has shown that a mutation in *FOLH1* that increases GCPII expression is associated with reduced NAAG brain levels measured in vivo by MRS, inefficient dlPFC activation during working memory, impaired visual memory and lower IQ scores [27]. MRS imaging has also shown reduced NAAG levels linked to cognitive deficits in patients with multiple sclerosis [26] and in those with dementia from AD [65, 66]. Although some reductions in NAAG may be due to lost neurons in AD, the finding that GCPII is associated with impaired cognition in healthy subjects emphasizes the importance of this signaling pathway to human intelligence.

Alterations in mGluR3-NAAG-GCPII signaling are consistently linked to schizophrenia, where mutations to *GRM3* (mGluR3) are a replicated risk factor by GWAS [18]. Insults that reduce mGluR3 expression and/or function are increasingly linked to impaired dlPFC cognitive abilities in schizophrenia [67]. Genetic variations in *GRM3* are associated with weaker dlPFC cognitive abilities, and altered activation of dlPFC during performance of cognitive tasks [60]. Postmortem brain studies have shown reduced mGluR3 and increased GCPII expression in the dlPFC of patients with schizophrenia [19]. Taken together, this work suggests that patients with schizophrenia may have much lower levels of mGluR3 stimulation in dlPFC [19], and thus may benefit from GCPII inhibition, as has been seen in rodent models [68, 69].

Reductions in NAAG from the association cortex have also been seen in AD, where decreased NAAG measured by MRS correlated with increased cognitive deficits in vivo [70]. Post-mortem assays have also documented reduced NAAG levels in the brains of patients with AD [23, 24]. As mGluR3 signaling regulates feedforward cAMP-calcium signaling in dlPFC, reductions in mGluR3 regulation may contribute to calcium dysregulation and the induction of AD tau pathology [71], similar to what is seen with severe COVID19 infection [13]. As GCPII inhibitors have a very low side effect profile [48], they may be helpful as a “baby aspirin” preventative approach for reducing risk of AD pathology.

Rodent studies have shown multiple benefits with GCPII inhibitors [72] in inflammatory disorders, including protecting cognition in models of stroke [30], MS [26], TBI [73], and AD [74]. Studies of cell cultures have also shown that mGluR3 is protective against AD pathology [75]. The beneficial effects of GCPII inhibitors appear to arise from boosting mGluR3 stimulation, as the enhancing effects of a GCPII inhibitor on recognition memory were not evident in mGluR3 knockout mice [74]. These results are consistent with the finding that mGluR3 stimulation is necessary for hippocampal-dependent meta-plasticity [76], indicating that mGluR3 stimulation is beneficial to hippocampal memory functions. We have just shown that systemic treatment with 2-MPPA can improve working memory in adult and aged rats [50]. Thus, there is a substantial literature in rodents on the benefits of GCPII inhibition for cognitive function. However, the current study is the first to show cognitive improvement with GCPII inhibition in primates, where the post-synaptic role of mGluR3 signaling greatly expands in the recently evolved circuits mediating higher cognition. The marked enhancement of dlPFC neuronal firing following direct application of either 2-MPPA or 2-PMPA emphasizes the importance of this mechanism to higher cognitive operations, boosting beneficial mGluR3 regulation in vulnerable dlPFC circuits, and encourages the development of GCPII inhibitors for human use.

## Supplementary information

Supplementary Material for Yang, Datta et al.
