# Supplementary material for: Inhibition of glutamate-carboxypeptidase-II in dorsolateral prefrontal cortex: potential therapeutic target for neuroinflammatory cognitive disorders
Source: Mol Psychiatry. Author manuscript; Available in PMC 2022 Dec 6. (PMC9718677; doi:10.1038/s41380-022-01656-x)
Supplement: GCPII supplementary material [file NIHMS1834078-supplement-GCPII_supplementary_material.docx]

**SUPPLEMENTARY MATERIAL**

**Figure S1**- **The traditional spatial delayed response task as performed in a Wisconsin General Test Apparatus.** In this task, the monkey watches as the experimenter bait one of two wells with a food reward, the wells are then covered with identical plaques and a screen is lowered for a prescribed delay. After the delay period is over, the screen is raised and the monkey must choose based on its memory of the location of the baited well. The spatial position of the reward randomly changes over the 30 trials that make up a daily test session, and the monkey must constantly update the contents of working memory to perform correctly.

**
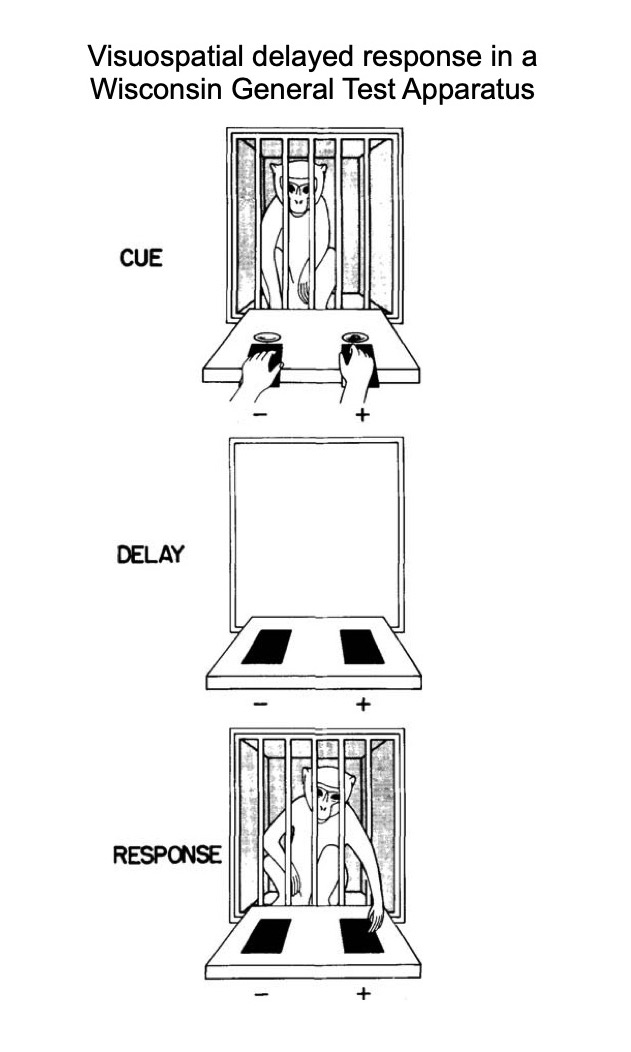
**

**Figure S2**- **GCPII immunolabeling by immunohistochemistry in aged macaque dlPFC.**

**(a-b)** High-magnification micrographs showing immunoperoxidase labeling for GCPII in aged macaque (28-30y) dlPFC layer III expression in multiple cell-types, including neurons with pyramidal cell-like characteristics including triangular shaped cell bodies and well-defined apical dendrites. Scale bars: 10µm. **(c)** Low magnification immunoEM micrograph showing GCPII immunoperoxidase immunolabeling in a pyramidal neuron with a triangular shaped cell body in dlPFC deep layer III. The GCPII immunolabeling is observed in the cell soma and extending along the apical dendrite (traced in magenta). GCPII protein is also observed in the neuropil. Scale bar: 2µm. **(d)** Low-magnification micrograph showing GCPII immunolabeling across cortical layers in aged rhesus macaque dlPFC.


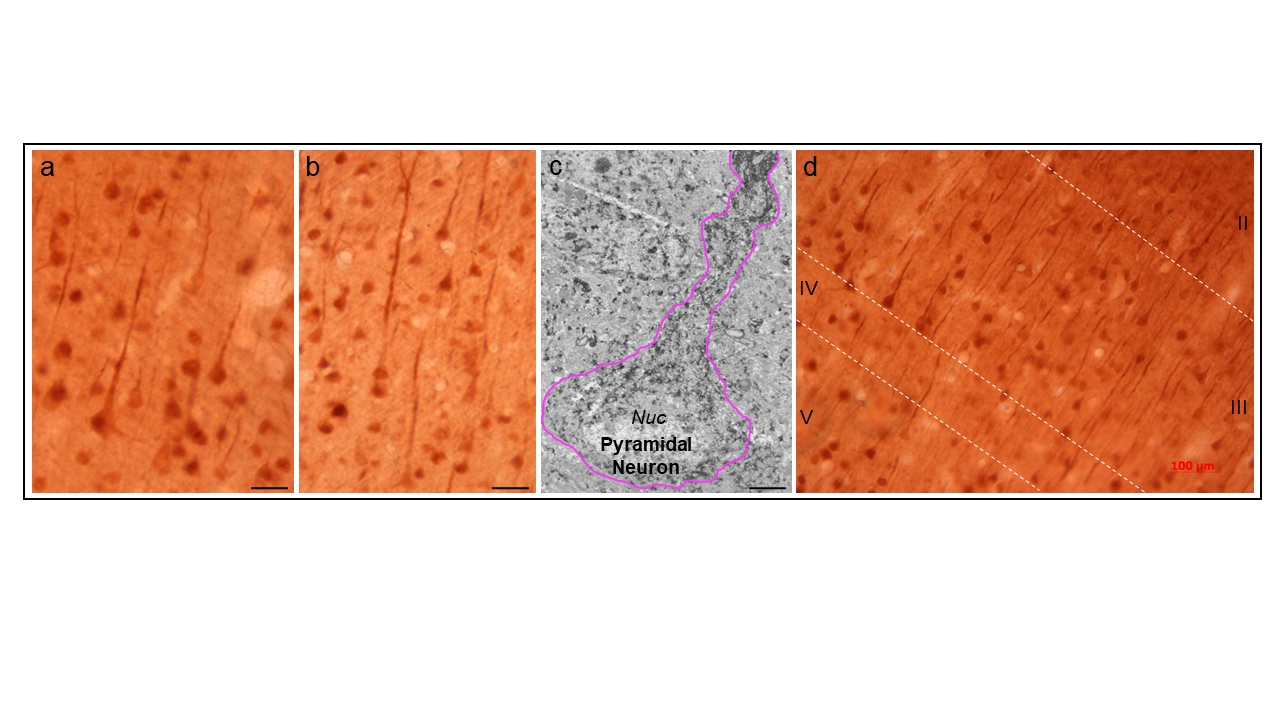


**Figure S3-** Analyses of GCPII inhibitor effects on Delay cell firing during the intertrial interval (ITI), the fixation epoch when the monkey first initiates a trial by fixating on the central point, and the cue, delay and response epochs. Both 2-MPPA and 2-PMPA increased task-related firing, but did not increase firing during the ITI. **(A)** The effects of 2-MPPA on Delay cell firing. 2-MPPA produced significant increase in cue-, delay- and response-epoch firing (paired t test, control vs 2-MPPA, cue epoch, p=0.0123; delay epoch, p=0.0029; response epoch, p=0.0026) as well as non-significant increase in ITI and fixation epoch firing (ITI, p=0.0543; fixation epoch, p=0.113). Further analysis indicated that 2-PMPA produced a greater increase in delay firing than ITI and fixation epochs (2-way ANOVA-R, Delay vs ITI, p=0.033; Delay vs Fixation, p=0.0066; Delay vs Cue, p=0.42; Delay vs Response, p=0.6221). **(B)** The effects of 2-PMPA on Delay cell firing. 2-PMPA produced significant increase in cue-, delay- and response-epoch firing (paired t test, control vs 2-PMPA, cue epoch, p=0.0243; delay epoch, p=0.0038; response epoch, p=0.0149) as well as non-significant increase in ITI and fixation epoch firing (ITI, p=0.2398; fixation epoch, p=0.0595). Further analysis indicated that 2-PMPA produced a greater increase in delay firing than ITI, fixation and response epochs (2-way ANOVA-R, Delay vs ITI, p=0.016; Delay vs Fixation, p=0.041; Delay vs Cue, p=0.4629; Delay vs Response, p=0.0267).

**
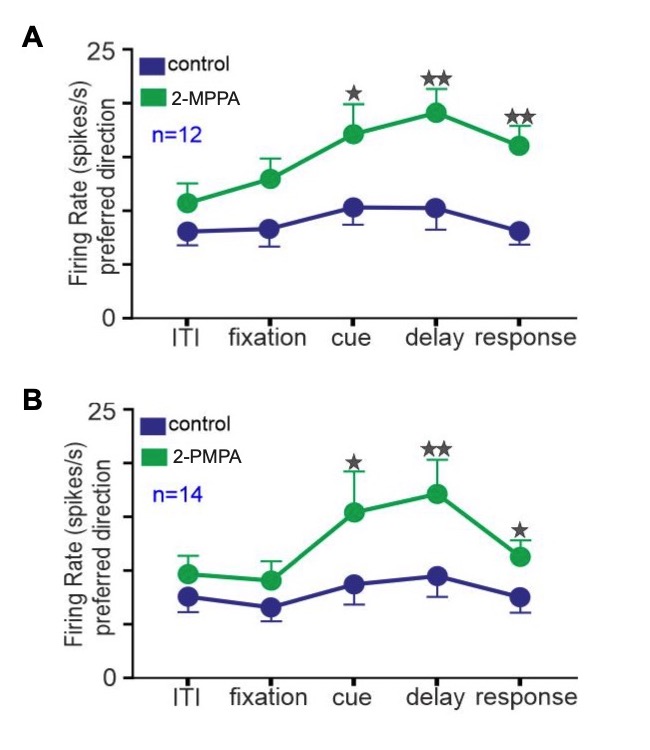
**
